# Subcutaneous versus intravenous ultra-low-dose rituximab in rheumatoid arthritis: a randomized controlled PK-PD non-inferiority trial

**DOI:** 10.1093/rheumatology/keag420

**Published:** 2026-08-18

**Authors:** Pauline M E T Bovens, Céleste J T van der Togt, Nathan den Broeder, Lise M Verhoef, Aatke van der Maas, Willemijn Hobo, Karien Bloem, Rob ter Heine, Bart J F van den Bemt, Alfons A den Broeder

**Affiliations:** Department of Rheumatology, Sint Maartenskliniek, Ubbergen, The Netherlands; Department of Rheumatology, Radboudumc, Nijmegen, The Netherlands; Department of Rheumatology, Radboudumc, Nijmegen, The Netherlands; Department of Research, Sint Maartenskliniek, Ubbergen, The Netherlands; Department of Research, Sint Maartenskliniek, Ubbergen, The Netherlands; Department of Rheumatology, Sint Maartenskliniek, Ubbergen, The Netherlands; Department of Laboratory Medicine, Laboratory of Hematology, Radboudumc, Nijmegen, The Netherlands; Laboratory, PurSang Diagnostiek, Biologics and Immunopathology, Amsterdam, The Netherlands; Department of Pharmacy, Pharmacology & Toxicology, Radboud University Medical Center, Nijmegen, The Netherlands; Department of Research, Sint Maartenskliniek, Ubbergen, The Netherlands; Department of Pharmacy, Pharmacology & Toxicology, Radboud University Medical Center, Nijmegen, The Netherlands; Department of Pharmacy, Sint Maartenskliniek, Nijmegen, The Netherlands; Department of Rheumatology, Sint Maartenskliniek, Ubbergen, The Netherlands; Department of Rheumatology, Radboudumc, Nijmegen, The Netherlands

**Keywords:** rituximab, subcutaneous, rheumatoid arthritis, non-inferiority, bioequivalence

## Abstract

**Objectives:**

Ultra-low-dose rituximab is an effective treatment in RA. S.c. administration may further facilitate rituximab treatment, reduce infection risk and reduce costs. This study aimed to assess non-inferiority of s.c. *vs* i.v. rituximab regarding pharmacokinetic and pharmacodynamic outcomes.

**Methods:**

In this randomized, open-label, non-inferiority study, RA patients with stable low disease activity on ultra-low-dose rituximab (500 mg or 200 mg every 6 months) were 1:1 randomized to receive rituximab 336 mg s.c. or 200 mg i.v., stratified by previous rituximab dose. Primary outcome was non-inferiority of s.c. to i.v. rituximab, based on the estimated rituximab serum concentration area under the curve during the first 6 months (AUC_0–6m_), with a non-inferiority margin of 0.8. AUCs were estimated using PK modelling. Secondary outcomes included change in DAS28-CRP compared with a non-inferiority margin of 0.6.

**Results:**

Thirty-five patients were randomized (17 i.v., 18 s.c.). The geometric mean ratio for AUC_0–6m_ was 0.99 (90% CI 0.82–1.20), with minimal bias [mean predication error: i.v. 13.1 (1.86%); s.c. −3.40 (−0.47%)] but moderate random prediction error [root mean square error: i.v. 140 (19.8%); s.c. 142 (19.5%)] for the AUC estimates. Mean change in DAS28-CRP was non-inferior for s.c. rituximab (−0.39, 95% CI −0.77 to −0.004).

**Conclusion:**

Our data suggest non-inferior pharmacokinetics for s.c. rituximab; however, this cannot be definitively concluded due to uncertainty of the AUC estimates. Nevertheless, based on pharmacokinetic and pharmacodynamic parameters, ultra-low-dose s.c. rituximab could be an alternative for ultra-low-dose i.v. rituximab.

Rheumatology key messages

- Based on pharmacokinetic/pharmacodynamic parameters and clinical outcome, ultra-low-dose s.c. rituximab could be an alternative for RA patients.
- Patients who received s.c. rituximab preferred s.c. injection over i.v. administration.

## Introduction

Rituximab is an effective and safe treatment for patients with RA. Rituximab, an mAb targeting CD20 on the surface of B cells, was originally developed as treatment for non-Hodgkin’s lymphoma. Since no formal dose finding studies have been performed for the registration of rituximab in RA, the recommended dosing schedule of 2 × 1000 mg i.v. (given 2 weeks apart) is based on the schedule for non-Hodgkin’s lymphoma [1]. However, the dosage of rituximab in standard practice for inflammatory diseases has been lowered substantially the last few years, optimizing the treatment with rituximab in patients with RA [2].

Several studies have shown that lower rituximab doses in RA are also effective and safe. A systematic review and meta-analysis by Bredemeier *et al.* compared low-dose rituximab (2 × 500 mg given 2 weeks apart or 1 × 1000 mg every 6 months) with high-dose rituximab (2 × 1000 mg given 2 weeks apart every 6 months) in RA patients, and low-dose rituximab proved to be non-inferior to high-dose rituximab [3]. A randomized controlled trial by Verhoef *et al.* examined the efficacy of ultra-low-dose rituximab (1 × 500 mg or 1 × 200 mg every 6 months) compared with low-dose (1 × 1000 mg every 6 months) (Ultra-low doses of rituximab for continued treatment of rheumatoid arthritis (REDO study)) for continued treatment. This study, and its long-term observational extension, showed that ultra-low-dose rituximab was an effective treatment option for a majority of RA patients responding well to standard-dose rituximab [4, 5].

Lower doses of rituximab offer several benefits. First, they are associated with fewer adverse events, including a significantly lower risk of infections [4]. Additionally, they provide a lower risk for infusion-related reactions and incident hypogammaglobulinaemia, and an improved response tovaccination, including COVID-19 [3, 4, 6, 7]. Moreover, the medication costs are substantially lower. Finally, the infusion time is shorter, reducing the time patients spend in the hospital and further contributing to cost reduction.

To further optimize rituximab treatment in RA patients, s.c. administration could be explored. A systematic review by Durand *et al.* shows that most RA patients on biological DMARD therapy prefer s.c. administration over i.v. administration, because it allows self-administration, and therefore increases self-management [8, 9]. Other benefits of s.c. administration are fewer hospital visits for patients, fewer clinical staff resources required and lower costs, making it a more sustainable treatment option [10].

An s.c. solution of rituximab is available and authorized for the treatment of follicular non-Hodgkin’s lymphoma [11, 12]. In the phase II and III studies, it was found that a fixed dose of 1400 mg s.c. rituximab every 2–3 months had non-inferior serum C_trough_ levels compared with 375 mg/m^2^ i.v. rituximab [13]. The bioavailability of this s.c. rituximab formulation was estimated at 63–65% [13, 14]. However, there are no studies investigating lower dosages of s.c. rituximab, nor any studies of s.c. rituximab in patients with RA. Therefore, it remains unclear whether ultra-low-dose s.c. rituximab is non-inferior to ultra-low-dose i.v. rituximab.

The aim of this study was therefore to investigate non-inferiority of ultra-low-dose s.c. rituximab to ultra-low-dose i.v. rituximab regarding pharmacokinetic (PK) and pharmacodynamic outcomes in patients with RA. Furthermore, we examined the PK properties, pharmacodynamic effects such as disease activity and effect on B cells, and patient preferences concerning s.c. rituximab compared with i.v. rituximab.

## Patients and methods

### Study design and participants

This study was a randomized, open-label, parallel, non-inferiority trial performed at the rheumatology department of the Sint Maartenskliniek, Nijmegen, The Netherlands between May 2022 and September 2024. Inclusion criteria were comparable to the REDO-study [4]. Patients were found eligible if they were above 16 years of age; had a diagnosis of RA, according to the 2010 EULAR/ACR criteria or a clinical diagnosis from a treating rheumatologist; were currently treated with ultra-low-dose rituximab (200 mg or 500 mg, with the possibility to taper to 200 mg, every 6 months), with or without concomitant conventional synthetic DMARD; and had a sufficient response to rituximab, defined as DAS28-CRP <2.9 for ≥3 months after the last infusion or low disease activity as judged by a treating physician. Patients were excluded if they had non-response to ultra-low-dose rituximab, defined as DAS28-CRP >2.9, or had a contraindication to either treatment option. Eligible patients were invited to participate in the study with approval of their treating physician, shortly before their next rituximab infusion. All participants provided written informed consent. Ethical approval was obtained from the local ethics committee (METC Oost-Nederland, NL74149.091.20). The study was registered in the Dutch trial register (NL-OMON52719).

### Randomization

After providing informed consent, patients were randomly assigned to either s.c. or i.v. rituximab in a 1:1 ratio. Randomization was done by a computerized procedure using Castor EDC. Random block sizes (sizes 2–4) were used to decrease the predictability of the allocation. Allocation was stratified by previous rituximab dose, either 500 mg or 200 mg, to account for potential pre-dose effects on post-dose kinetics. The allocation was revealed to patients, care providers and the research team after randomization.

### Procedures

Patients received rituximab according to their allocated administration route at the start of the study. Patients in the intervention group received a single dose of 336 mg rituximab s.c. (MabThera®, Roche BV, solution for s.c. injection 120 mg/ml), administered by a qualified nurse in the abdominal skin. No premedication was administered, based on a retrospective study by Burrows *et al.* showing that s.c. rituximab can be safely administered without premedication in patients with lymphoma [15].

The s.c. dose of 336 mg was chosen based on the known bioavailability of 63%, implying that a s.c. dose of 317 mg would be sufficient to equal 200 mg i.v. [14]. However, as the only available formula of s.c. rituximab contains 1400 mg rituximab in 11.7 ml, we pragmatically chose to divide the solution for injection over four patients, each receiving 2.8 ml accounting for 0.5 ml spillage by filling of the syringes.

Patients in the control group received 200 mg i.v. rituximab (Rixathon®, Sandoz BV, solution for infusion 10 mg/ml) with standard premedication according to local protocol (50 mg prednisolone orally, 1000 mg paracetamol orally and 10 mg cetirizine orally).

Follow-up visits with a nurse or physician were planned at 3 and 6 months after start of the study. Extra visits were planned in case of a flare-up, defined as an increase in disease activity (DAS28-CRP >2.9 or increase of >1.2). A flare was first treated with glucocorticoids (oral, IA or i.m.) and, when necessary, an extra infusion with 200 mg rituximab i.v. could be given. The aim was to keep all background medication unaltered during the study and to document any changes.

Data on demographics, disease and treatment characteristics, quality of life and disease activity were collected at baseline. In addition, blood samples for PK [rituximab serum levels and anti-drug antibodies (ADA)] were collected pre-dose, and directly after infusion or 3–4 days after s.c. injection. B-cell levels were measured before rituximab administration. At the 3- and 6-month visits, disease activity, rituximab-levels, ADA, B-cell levels and quality of life were assessed. Adverse events were monitored throughout the study.

### Sample analysis

#### Rituximab levels

Serum concentrations of rituximab were quantified with a validated ELISA assay at the biologics laboratory of Sanquin Diagnostic Services (Amsterdam, The Netherlands). Rituximab was captured by rabbit-anti-rituximab bound to the ELISA plate. The bound rituximab was detected by biotinylated rabbit‐anti‐rituximab F(ab′)_2_ fragments, followed by poly-HRP‐labelled streptavidin and TMB. The signal was back calculated to a concentration using a standard curve of rituximab. The range of the assay was 0.005–500 mg/l [16].

#### Anti-drug antibodies

The presence of ADA against rituximab was measured and semi-quantified in the serum of patients with low rituximab levels (<0.5 mg/l). Samples were analysed using a validated ADA radioimmune assay at the biologics laboratory of Sanquin Diagnostic Services. Shortly, all antibodies were captured using protein A Sepharose. ADA against rituximab were detected using F(ab′)_2_ fragments of rituximab in combination with radiolabelled streptavidin. Signals were compared with a standard curve of a rabbit polyclonal ADA pool against rituximab. ADA were considered positive if above the cut-off of 8 AU/ml and are semi-quantifiable above 30 AU/ml [16].

#### B-cell count

Peripheral B cells were measured by a validated flow cytometric diagnostic assay at the Radboudumc Haematology laboratory. The percentages and absolute number of B cells were determined within 24 h after blood collection by using a combination of fluorochrome-labelled mAbs, CD45, CD3, CD19 and CD16/56 (Tetra 2 kit, Beckman Coulter). With the use of a fully standardized and validated volumetric counting flow cytometer, the Aquios (Beckman Coulter), samples were incubated with mAbs, erythrocytes were lysed, and subsequently the analysis was automatically performed. Samples were calibrated using standardized fixed cells samples (Aquios Immuno-Trol cells, Beckman Coulter). External quality control assessment was performed by participating in the regular program of the UK Neqas (Sheffield), the Alternative Immunomonitoring Program (12 samples per year).

### Outcomes

The primary outcome was non-inferiority of the rituximab serum concentration area under the curve over 6 months (AUC_0–6m_) of s.c. rituximab compared with i.v. rituximab. S.c. rituximab was considered non-inferior to i.v. rituximab if the lower boundary of the 90% CI of the ratio of AUC_0–6m_ in the s.c. group compared with the i.v. group exceeded the non-inferiority margin of 0.8. In contrast to the clinical trials involving s.c. rituximab in patients with non-Hodgkin’s lymphoma, we chose AUC_0–6m_ as primary endpoint instead of trough levels (C_trough_) [13]. We assume rituximab exposure over time, as reflected by the AUC_0–6m_, is more relevant in patients with RA than C_trough_ due to (i) a weaker correlation between C_trough_ and therapeutic efficacy, given persistent disease control despite lower trough levels due to longer dosing intervals; and (ii) a different pharmacodynamic profile in which the therapeutic goal is reduced inflammatory activity instead of complete B-cell depletion. The empirical Bayes estimates for the AUCs were obtained by non-linear mixed effects modelling, see the ‘Statistical analysis’ section. This was an adaptation of the original protocol, in which we specified that AUC_0–6m_ would be calculated using the trapezoidal method. However, in hindsight we deemed the sampling scheme too sparse to accurately determine the AUC_0–6m_ with the trapezoid method. Comparison of the AUCs calculated with the trapezoidal method was added as a secondary outcome. Other secondary outcomes included other PK parameters: peak level and trough level; disease activity, measured by DAS28-CRP; B-cell counts; patient preferences (s.c. *vs* i.v. injection); and adverse events, graded according to the Common Terminology Criteria for Adverse Events version 5. Assessment of quality of life, measured by the EuroQol-5D-5L questionnaire [17], and measurement of ADA were added as protocol amendments after the initial approval of the study protocol.

### Statistical analysis

#### Sample size

The sample size was determined based on a one-sided *t*-test. A non-inferiority margin of 0.8 was chosen based on the standard bioequivalence range used by the European Medicines Agency for bioequivalence studies [18]. Assuming that the AUC of 200 mg i.v. and 336 mg s.c. are equal, a coefficient of variation in AUC of 25% was calculated from the serum rituximab levels from the REDO-study [3]. Based on an alpha of 5% and a power of 80%, 18 patients in each group were included to show non-inferiority, accounting for 5% drop-out.

#### Primary outcome/PK analysis

The empirical Bayes estimates for rituximab PK were obtained from the PK data in our study by means of non-linear mixed effects modelling, using the software package NONMEM V7.5 (Icon, Ireland). For this purpose, the population PK model for i.v. PK of rituximab in RA patients [19] was extended with the s.c. absorption bioavailability and rate constant as described previously for this drug in another population [14], under the assumption that indication does not impact s.c. drug disposition. The theoretical steady-state AUC between doses (6 month interval) was then calculated using the equation AUC =  (DOSE * F)/CL, where dose is the individual dose, F is the empirical bayes estimate for bioavailability (1 for i.v. administration and <1 for s.c. administration) and CL is the empirical bayes estimate for clearance. Since the dose interval is 6 months, the result of this calculation equals the AUC_0–6m_ at steady-state.

In order to compare AUC_0–6m_, s.c.:AUC_0–6m_, i.v. for all randomized patients, the geometric mean ratio (GMR) was estimated using an analysis of variance model fitted on log-transformed AUCs, with allocation and stratification factor included as fixed effects. This model was also used to compare the AUCs calculated by the trapezoid method for complete cases.

To evaluate the bias and precision of the AUC estimation, the study design was simulated 15 times, generating ∼500 virtual individuals. This allowed for a comparison between simulated (true) and estimated AUCs, and calculation of the mean precision error (MPE) and the root mean square error (RMSE). MPE and RMSE were reported as absolute values and percentages relative to the mean AUC.

#### Secondary outcomes

Change in disease activity from baseline was compared using an analysis of covariance test with administration route as the determinant, adjusted for stratum (previous rituximab dose) and baseline DAS28-CRP. Between-group difference in changes in disease activity was compared with a non-inferiority margin of 0.6. Missing DAS28-CRP values were imputed with multiple imputation. Other secondary outcomes were compared using linear regression, *t*-test, Mann–Whiney *U* test, Chi-squared test or Fisher’s exact test, depending on the data type and distribution. A two-sided *P*-value of <0.05 was considered statistically significant. All analyses were done using R (v4.4.0) and R studio (v4.1).

## Results

### Participants

**Figure 1:**
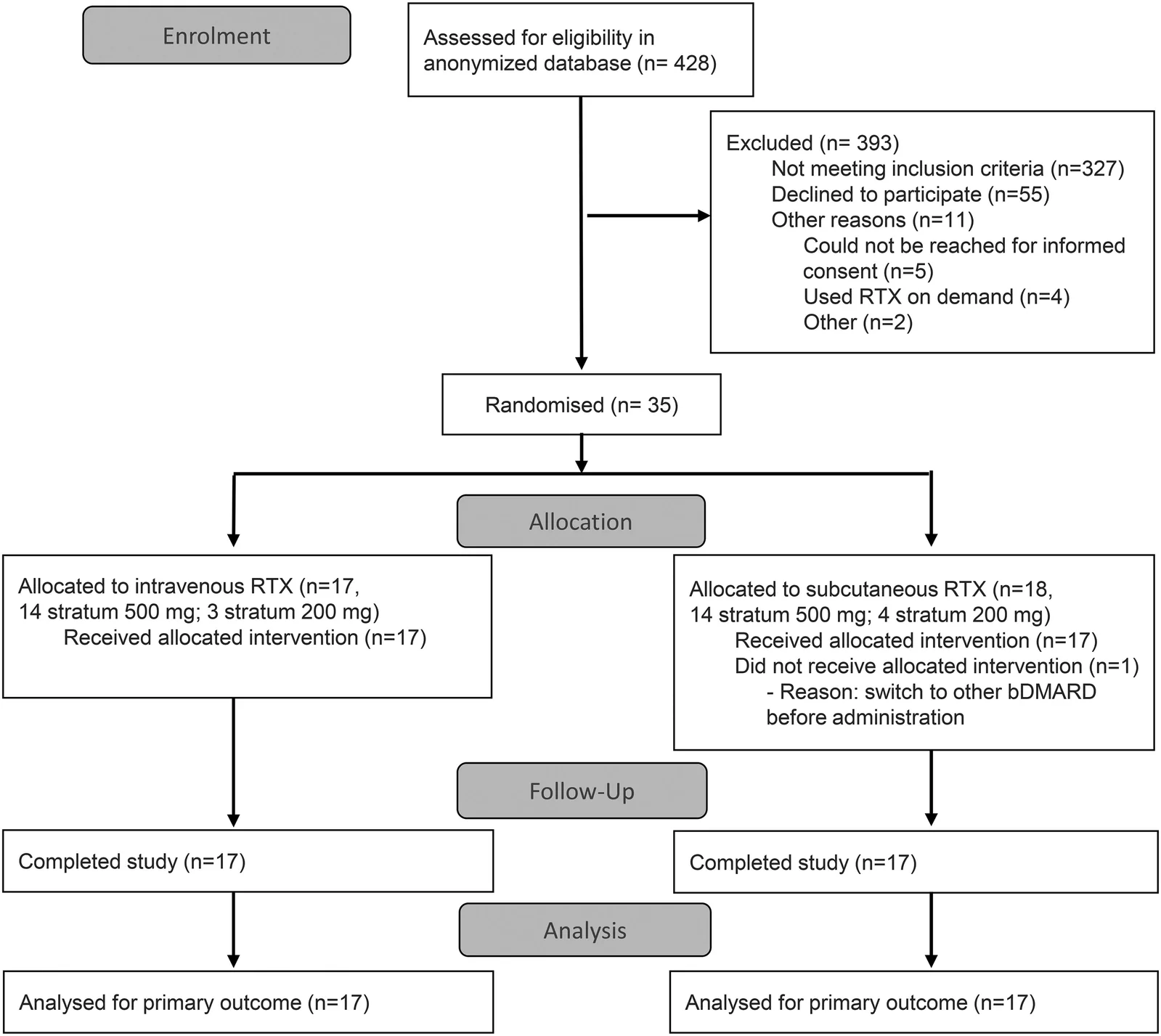
Flow diagram. All patients with an RA diagnosis and using rituximab were assessed for eligibility. Most patients not meeting the inclusion criteria were on 1000 mg or 500 mg rituximab and unable to taper to 200 mg

Patient recruitment started in May 2022 and the last visit was in September 2024. In total, 35 patients were randomized between the two administration groups; 18 were allocated to s.c. rituximab, and 17 were allocated to i.v. rituximab. One patient in the s.c. group switched to another biological DMARD before administration of rituximab and was therefore excluded from the study. All other participants completed the 6-month follow-up period (Fig. 1). Patient characteristics at baseline are comparable between the randomization groups and are presented in Table 1.

**Table 1:** Baseline characteristics of patients included in the analysis, by administration group.

	Subcutaneous (*N* = 17)	Intravenous (*N* = 17)
Age (years)	62 (12)	69 (9)
Female, *n* (%)	12 (71)	12 (71)
Disease duration (years)	17 (9)	17 (10)
RF and/or ACPA positive, *n* (%)	16 (94)	15 (88)
Rituximab duration (years)	5 (3–7)	6 (3–7)
Latest rituximab dosage, *n* (%)		
200 mg	13 (76)	14 (82)
500 mg	4 (24)	3 (18)
Concomitant use of csDMARD, *n* (%)	12 (71)	12 (71)
MTX	6 (50)	5 (42)
SSZ	5 (42)	4 (33)
LEF	0 (0)	1 (8.3)
Previous b/tsDMARDS	2 (1–2)	2 (1–2)
DAS28-CRP	1.92 (0.49)	1.79 (0.56)
Anti-drug antibodies against rituximab, *n* (%)	2 (11)	0 (0)

Data are presented as *n* (%), mean (s.d.) or median (interquartile range). csDMARD: conventional synthetic DMARD; bDMARD: biological DMARD; tsDMARD: targeted synthetic DMARD

### Primary outcome

**Figure 2:**
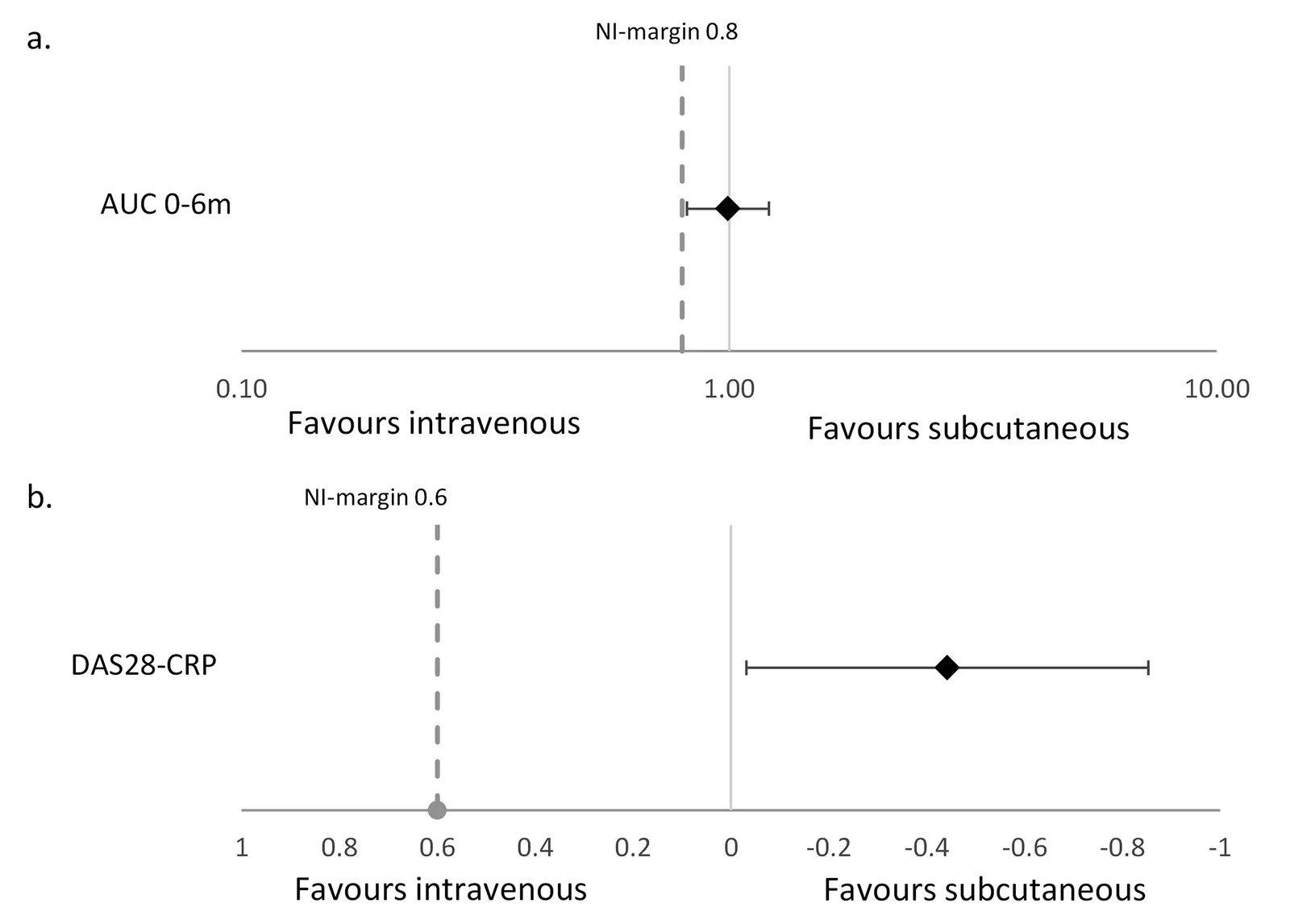
Comparing (a) geometric mean ratio of AUC_0–6m_ and (b) change of DAS28-CRP from baseline between s.c. rituximab and i.v. rituximab. Non-inferiority margin for AUC is 0.8 with a 90% CI, and for DAS28-CRP 0.6 with a 95% CI. AUC_0–6m_: area under the curve during the first 6 months

PK modelling of rituximab concentrations was used to estimate the AUC_0–6m_ of s.c. rituximab and i.v. rituximab for all randomized participants. Point estimates of the AUC_0–6m_ s.c. rituximab and AUC_0–6m_ i.v. rituximab were similar. The lower limit of the CI did not exceed the non-inferiority margin of 0.8 (GMR 0.99; 90% CI 0.82–1.20) (Fig. 2a). The bias of the AUC estimation was minimal at ∼1% [MPE: i.v. 13.1 (1.86%); s.c. −3.40 (−0.47%)]; however, the RMSE of the AUC estimation was ∼20% [i.v. 140 (19.8%); s.c. 142 (19.5%)], indicating moderate random prediction error. AUCs calculated by the trapezoidal method for complete cases (s.c.: *n* = 14; i.v. *n* = 11) were higher in both administration groups than the modelled AUCs (s.c. 1339 *vs* 744; i.v. 3302 *vs* 758, respectively), with the resulting GMR falling below the non-inferiority margin of 0.8 (GMR 0.40; 90% CI 0.32–0.49).

### Pharmacokinetics

For additional PK parameters, rituximab serum levels were compared between s.c. and i.v. administration. Rituximab levels directly after infusion or 2–6 days after s.c. injection were considered to approximate peak concentrations (C_peak_). However, for the s.c. injection the true peak may not have been captured due to off-peak sampling. C_peak_ was significantly lower for s.c. rituximab compared with i.v. rituximab (GMR 0.38; 95% CI 0.31–0.48). The rituximab trough levels (C_trough)_ were measured just before the following infusion, ∼6 months after rituximab administration/baseline. No significant difference in C_trough_ was observed between the two groups (GMR 0.78; 95% CI 0.27–2.31). Missing rituximab levels (s.c. *n* = 2; i.v. *n* = 2) were left out of the analyses; individual rituximab levels at different time points are presented in Supplementary Fig. S1.

### Pharmacodynamics

**Figure 3:**
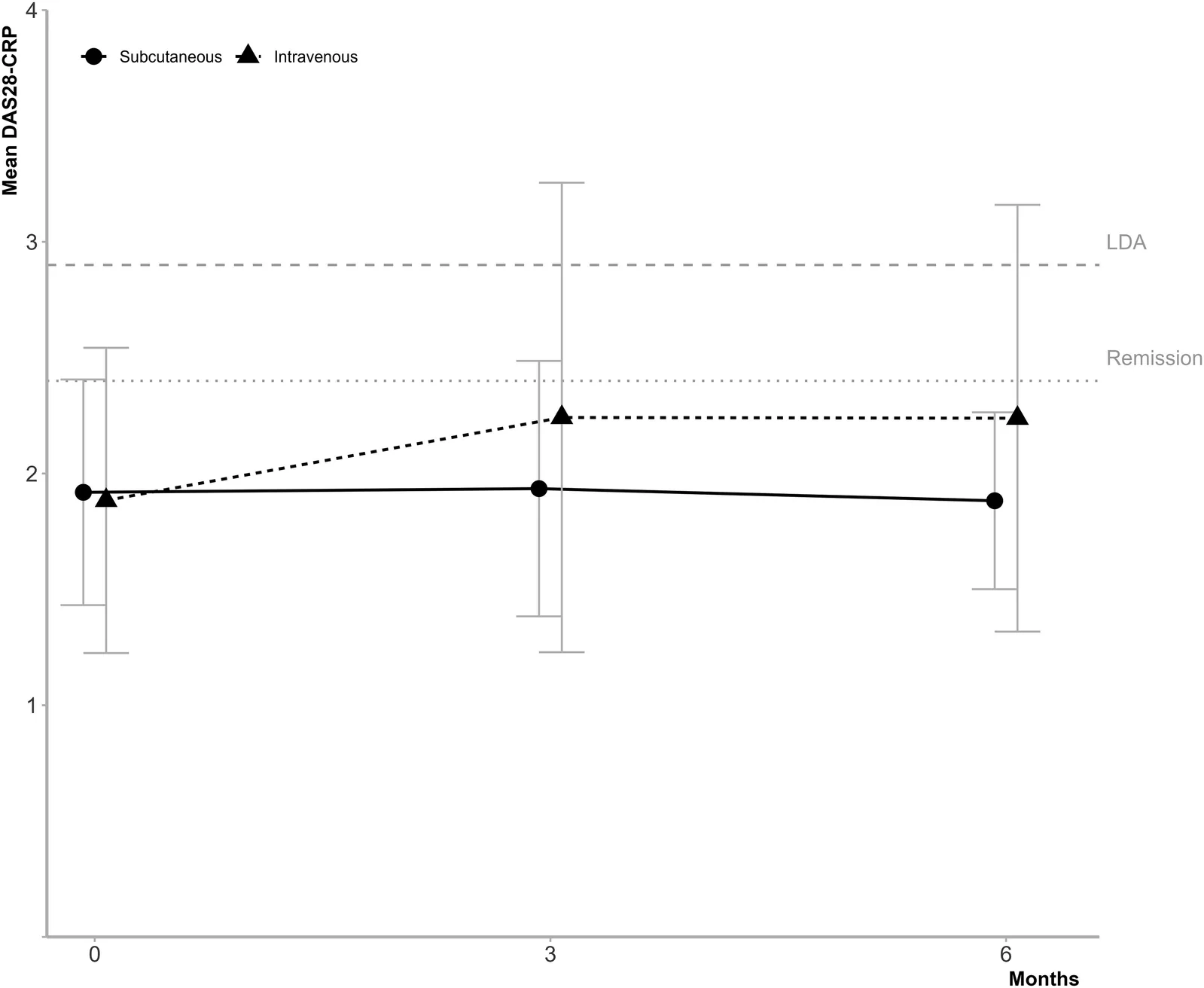
Comparing disease activity between s.c. and i.v. rituximab administration during the study. Disease activity was measured as DAS28-CRP, data represent means ± s.d. One DAS28-CRP measurement was missing at baseline and five at 6 months; these were imputed using multiple imputation. Remission was defined as DAS28-CRP <2.4 and low disease activity (LDA) as DAS28-CRP <2.9

**Figure 4:**
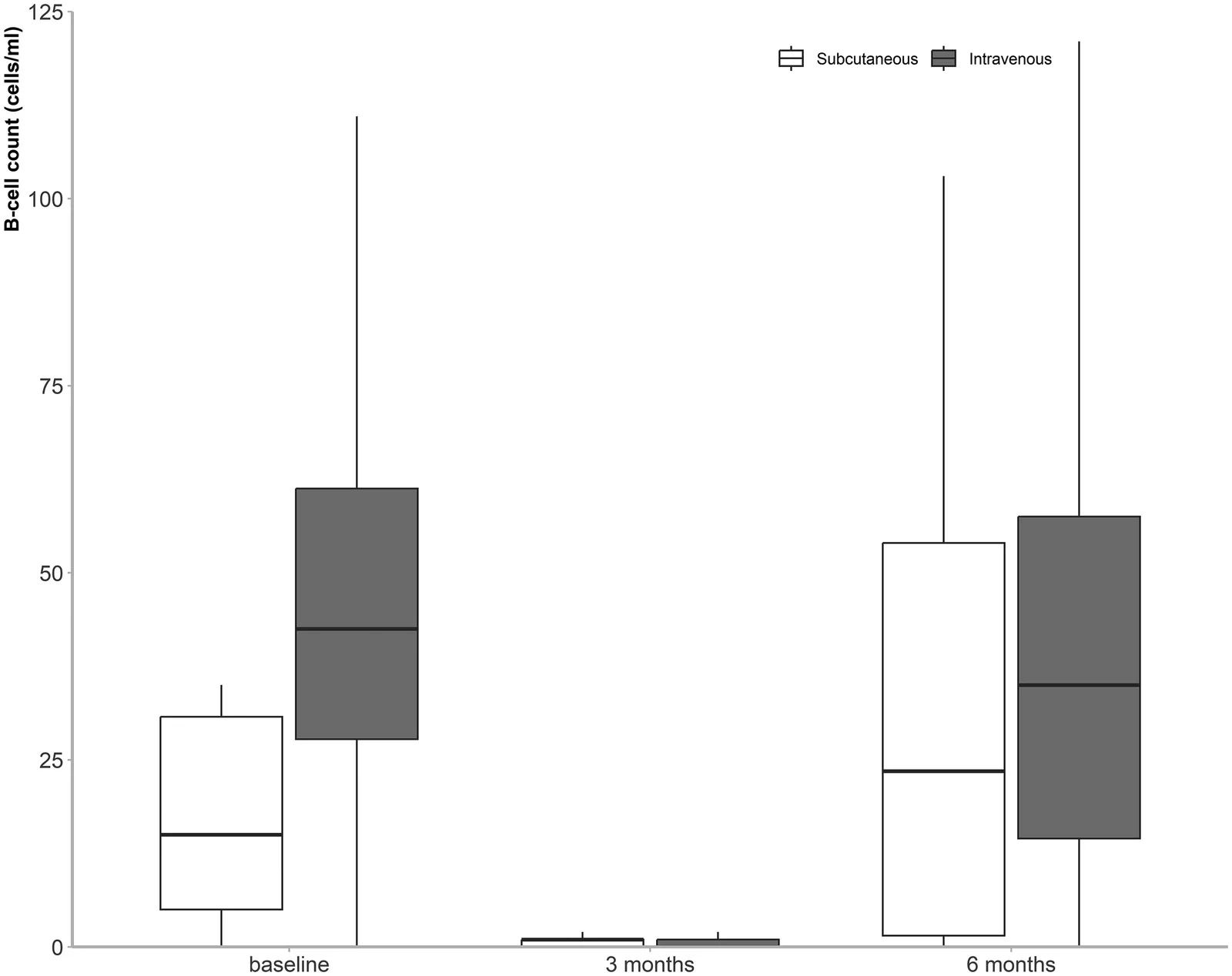
Median B-cell counts at baseline, and 3- and 6-month measurements. Six B-cell measurements were missing—two at baseline (1 s.c., 1 i.v.) and four at 6 months (2 s.c., 2 i.v.)—and were therefore not included in the analysis

Mean changes in DAS28-CRP were compared between s.c. and i.v. administration of rituximab (Fig. 3). DAS28-CRP in the i.v. administration group increased non-significantly from baseline, while there was minimal change in the s.c. group (adjusted mean difference −0.44, 95% CI −0.85 to −0.032). Since the 95% CI did not exceed the pre-specified limit of 0.6, s.c. rituximab is non-inferior to i.v. rituximab (Fig. 2b) with respect to DAS28-CRP. At the 3-month visit, three patients in the i.v. group had clinically active disease and one patient at the 6-month visit. In the s.c. group, one patient required an extra visit due to a flare. B-cell counts were taken at baseline, before rituximab administration, at 3 and 6 months. No significant differences in B-cell counts were found between s.c. or i.v. administration at any time point (baseline: *P *= 0.12; 3 months: *P* = 0.51; 6 months: *P* = 0.59) (Fig. 4). ADA against rituximab were measured in the serum of patients with low rituximab levels (<0.5 mg/l). At baseline, two patients had ADA against rituximab, both were allocated to s.c. rituximab in the study (Table 1). At 3 and 6 months, ADA were measured in respectively one and both of these patients. No ADA were measured in patients who received i.v. rituximab.

### Safety, quality of life and patient preference

The proportion of patients with adverse events was higher in the s.c. administration group [s.c. 12/17 (70.6%); i.v. 7/17 (41.2%), *P* = 0.40], mostly due to injection site reactions (7/17 patients; 41%) (Table 2). All injection site reactions were mild and resolved quickly. Two serious adverse events were reported. One patient in the i.v. group had a stroke and required hospitalization with full recovery. One patient in the s.c. group had a syncope and was hospitalized for 3 days without finding a cause. Both adverse events occurred around 2 months after rituximab administration. Of the total adverse events, infections were more common in the i.v. group (s.c. 6/18; i.v. 8/14). Most were mild respiratory infections. No difference in change of quality of life was observed between the groups (0.03, 95% CI −0.06–0.11) (Supplementary Table S1). Within the patients in the s.c. group, 13/17 (76%) preferred the s.c. injection.

**Table 2:** Adverse events.

	Overall (*N* = 32)	Subcutaneous (*N* = 18)	Intravenous (*N* = 14)
Category			
Infections and infestations	14 (44)	6 (33)	8 (57)
General disorders and administration site conditions	7 (22)	7 (39)	0 (0)
Nervous system disorders	3 (9.4)	1 (5.6)	2 (14)
Skin and s.c. tissue disorders	2 (6.3)	2 (11)	0 (0)
Vascular disorders	2 (6.3)	0 (0)	2 (14)
Eye disorders	1 (3.1)	0 (0)	1 (7.1)
Gastrointestinal disorders	1 (3.1)	1 (5.6)	0 (0)
Injury, poisoning and procedural complications	1 (3.1)	1 (5.6)	0 (0)
Musculoskeletal and connective tissue disorders	1 (3.1)	0 (0)	1 (7.1)
Grade			
1	20 (63)	13 (72)	7 (50)
2	8 (25)	3 (17)	5 (36)
3	4 (13)	2 (11)	2 (14)
SAE	2 (6.3)	1 (5.6)	1 (7.1)

Data are presented as *n* (%). SAE: serious adverse events.

## Discussion

To our best knowledge, this is the first study to examine s.c. use of ultra-low-dose rituximab in patients with RA. This study found comparable serum rituximab AUC. Furthermore, we found non-inferior clinical disease activity for ultra-low-dose s.c. rituximab compared with i.v. rituximab. Additionally, no differences in B-cell count, ADA development and quality of life during the study were observed between the two administration groups. Also, the safety profiles were comparable, except for mild injection site reactions in the s.c. group. Finally, s.c. rituximab was preferred by patients. Although formal non-inferiority for our primary outcome could not be demonstrated due to limited sampling, these findings support the hypothesis that ultra-low-dose s.c. rituximab could be a safe and effective alternative to ultra-low-dose i.v. rituximab for patients with RA.

An important limitation of this study is the limited sampling scheme. Calculating the AUC using the trapezoidal method as originally intended likely overestimated the AUC, particularly for s.c. administration, because our sampling scheme did not accurately capture the more gradual decline of the concentration–time curve expected after s.c. dosing. Therefore, PK modelling was used to better estimate the AUC. Bayesian estimation of the AUC was predicted to result in a minimal bias, yet with a moderate amount of random prediction error. As this prediction error is not reflected in the CI of the between-group difference in AUC_0–6m_, non-inferiority cannot be shown conclusively. Nevertheless, s.c. injection resulted in comparable point estimates of serum rituximab levels to i.v. administration. Moreover, adequate B-cell depletion and clinical disease control were achieved. Another limitation is the follow-up period of 6 months, limiting generalizability to continued treatment with ultra-low-dose rituximab. However, as patients started the next cycle with comparable rituximab levels, B-cell counts and disease activity, it seems plausible that similar results would be acquired in subsequent cycles. Additionally, this study only assessed ultra-low-dose rituximab, not standard (1000 mg) or authorized high dose (2 × 1000 mg). However, since PK non-inferiority of s.c. rituximab has been shown for high-dose (1400 mg) in non-RA patients, and our findings are consistent with non-inferiority for ultra-low-dose, we expect that these results can be extrapolated to other dosages in between [11]. Finally, there is a risk for bio-creep since our comparator is the result of multiple non-inferiority trials. However, we believe this risk to be limited since there was no relevant creep observed across studies. Meta-analyses of five studies comparing 2 × 1000 mg *vs* 1 × 1000 mg (or 2 × 500 mg) showed a mean DAS28 difference of 0.07 (−0.08–0.22) in favour of 2 × 1000 mg [3]. The subsequent REDO study showed a mean DAS28 difference between 1 × 1000 mg and 1 × 200 mg of −0.02 (−0.39–0.35) in favour of 200 mg rituximab [4]. With the current study showing a DAS28 between-group difference of −0.44 in favour of s.c., there are no signs of bio-creep regarding DAS28-CRP outcomes at 6 months. Nevertheless, a larger trial comparing 200 mg s.c. rituximab with the original dose of 2 × 1000 mg rituximab and placebo would provide additional value.

S.c. injection of ultra-low-dose rituximab has a number of potential advantages over i.v. administration. It may allow for self-administration, reducing hospital visits for patients. S.c. injection also provides a feasible option for improving healthcare and treatment of RA when there is a shortage of qualified personnel, especially in low-income countries or areas with limited access to hospitals. Although no formal cost-effectiveness analysis was carried out, costs may be reduced, as the additional expenses of the s.c. formula are balanced by the absence of costs for day-treatment facilities. Furthermore, this study showed that s.c. injection could be administered safely without the usual premedication given for i.v. rituximab in RA patients. However, an ultra-low-dose formula of s.c. rituximab is not available yet, limiting the implementation in standard care for RA patients.

## Conclusion

In conclusion, our data suggest non-inferior PK for ultra-low-dose s.c. rituximab in RA patients with stable low disease activity. However, this cannot be definitively concluded due to limited sampling being too sparse for precise assessment of the AUC. Nevertheless, ultra-low-dose s.c. rituximab might be an alternative for ultra-low-dose i.v. rituximab based on pharmacodynamic non-inferiority regarding disease activity. Since s.c. administration has many advantages, development of a low-dose s.c. rituximab formulation might enable potential implementation of these findings in standard care.

## Supplementary Material

keag420_Supplementary_Data

## Data Availability

The data underlying this article will be shared on reasonable request to the corresponding author.
