# Supplementary material for: Subcutaneous versus intravenous ultra-low-dose rituximab in rheumatoid arthritis: a randomized controlled PK-PD non-inferiority trial
Source: Rheumatology (Oxford). 2026 Aug 18;65(9):keag420. doi: 10.1093/rheumatology/keag420 (PMC13599780; doi:10.1093/rheumatology/keag420)
Supplement: Supplementary file 1 [file keag420_supplementary_data.docx]

Supplementary material

Table of contents

[Supplement 1. CONSORT checklist non-inferiority trials	2](#_Toc226984831)

[Supplement 2. Supplementary Table S1	4](#_Toc226984835)

[Supplement 3: Supplementary Figure S1	5](#_Toc226984836)

## Supplement 1. CONSORT checklist non-inferiority trials

CONSORT Statement 2006 - Checklist for Non-inferiority and Equivalence Trials

**Items to include when reporting a non-inferiority or equivalence randomized trial**

| ***PAPER SECTION* And topic** | **Item** | **Descriptor** | **Reported on**  **Page #** |
| --- | --- | --- | --- |
| *TITLE & ABSTRACT* | 1 | [How participants were allocated to interventions](http://www.consort-statement.org/index.aspx?o=1107) (*e.g*., "random allocation", "randomized", or "randomly assigned"),  s*pecifying that the trial is a non-inferiority or equivalence trial.* | page 1 & 3 |
| *INTRODUCTION* Background | 2 | [Scientific background and explanation of rationale](http://www.consort-statement.org/index.aspx?o=1016),  *including the rationale for using a non-inferiority or equivalence design.* | Page 4 & 5 |
| *METHODS* Participants | 3 | [Eligibility criteria for participants](http://www.consort-statement.org/index.aspx?o=1017#3a) *(detailing whether participants in the non-inferiority or equivalence trial are similar to those in any trial(s) that established efficacy of the reference treatment)* and the [settings and locations where the data were collected](http://www.consort-statement.org/index.aspx?o=1017#3b). | Page 5 & 6 |
| Interventions | 4 | [Precise details of the interventions intended for each group *detailing whether the reference treatment in the non-inferiority or equivalence trial is identical (or very similar) to that in any trial(s) that established efficacy,* and how and when they were actually administered](http://www.consort-statement.org/index.aspx?o=1021). | Page 6 |
| Objectives | 5 | [Specific objectives and hypotheses](http://www.consort-statement.org/index.aspx?o=1022), *including the hypothesis concerning non-inferiority or equivalence*. | Page 8 |
| Outcomes | 6 | [Clearly defined primary and secondary outcome measures](http://www.consort-statement.org/index.aspx?o=1023#6a) *detailing whether the outcomes in the non-inferiority or equivalence trial are identical (or very similar) to those in any trial(s) that established efficacy of the reference treatment* and, when applicable, any [methods used to enhance the quality of measurements](http://www.consort-statement.org/index.aspx?o=1023#6b) (*e.g.*, multiple observations, training of assessors). | Page 8 |
| Sample size | 7 | [How sample size was determined](http://www.consort-statement.org/index.aspx?o=1024#7a) *detailing whether it was calculated using a non-inferiority or equivalence criterion and specifying the margin of equivalence with the rationale for its choice*. When applicable, [explanation of any interim analyses and stopping rules](http://www.consort-statement.org/index.aspx?o=1024#7b) (*and whether related to a non-inferiority or equivalence hypothesis*). | Page 9 |
| Randomization -- Sequence generation | 8 | [Method used to generate the random allocation sequence, including details of any restrictions](http://www.consort-statement.org/index.aspx?o=1025) (*e.g*., blocking, stratification) | Page 6 |
| Randomization -- Allocation concealment | 9 | [Method used to implement the random allocation sequence](http://www.consort-statement.org/index.aspx?o=1026) (*e.g*., numbered containers or central telephone), clarifying whether the sequence was concealed until interventions were assigned. | Page 6 |
| Randomization -- Implementation | 10 | [Who generated the allocation sequence, who enrolled participants, and who assigned participants to their groups](http://www.consort-statement.org/index.aspx?o=1027). | Page 5 & 6 |
| Blinding (masking) | 11 | [Whether or not participants, those administering the interventions, and those assessing the outcomes were blinded to group assignment](http://www.consort-statement.org/index.aspx?o=1028#11a). If done, [how the success of blinding was evaluated](http://www.consort-statement.org/index.aspx?o=1028#11b). | Page 6 |
| Statistical methods | 12 | [Statistical methods used to compare groups for primary outcome(s)](http://www.consort-statement.org/index.aspx?o=1029#12a), *specifying whether a one or two-sided confidence interval approach was used*. [Methods for additional analyses](http://www.consort-statement.org/index.aspx?o=1029#12b), such as subgroup analyses and adjusted analyses. | Page 9 & 10 |
| *RESULTS*  Participant flow | 13 | [Flow of participants through each stage](http://www.consort-statement.org/index.aspx?o=1018) (a diagram is strongly recommended). Specifically, for each group report the numbers of participants randomly assigned, receiving intended treatment, completing the study protocol, and analyzed for the primary outcome. [Describe protocol deviations from study as planned, together with reasons](http://www.consort-statement.org/index.aspx?o=1086). | Figure 1 & page 10 |
| Recruitment | 14 | [Dates defining the periods of recruitment and follow-up](http://www.consort-statement.org/index.aspx?o=1087). | Page 10 |
| Baseline data | 15 | [Baseline demographic and clinical characteristics of each group](http://www.consort-statement.org/index.aspx?o=1088). | Page 10 & Table 1 |
| Numbers analyzed | 16 | [Number of participants (denominator) in each group included in each analysis and whether the analysis was](http://www.consort-statement.org/index.aspx?o=1089) *“intention-to-treat”* *and/or* *alternative analyses were conducted*. State the results in absolute numbers when feasible (*e.g*., 10/20, not 50%). | Page 10, 11, 12 |
| Outcomes and estimation | 17 | [For each primary and secondary outcome, a summary of results for each group, and the estimated effect size and its precision](http://www.consort-statement.org/index.aspx?o=1090) (*e.g.*, 95% confidence interval). *For the outcome(s) for which non-inferiority or equivalence is hypothesized, a figure showing confidence intervals and margins of equivalence may be useful*. | Page 10, 11, 12 |
| Ancillary analyses | 18 | [Address multiplicity by reporting any other analyses performed](http://www.consort-statement.org/index.aspx?o=1091), including subgroup analyses and adjusted analyses, indicating those pre-specified and those exploratory. | Page 10 & 11 |
| Adverse events | 19 | [All important adverse events or side effects in each intervention group](http://www.consort-statement.org/index.aspx?o=1092). | Page 12 & Table S1 |
| *DISCUSSION* Interpretation | 20 | [Interpretation of the results](http://www.consort-statement.org/index.aspx?o=1019), taking into account the *non-inferiority or equivalence hypothesis and any other* study hypotheses, sources of potential bias or imprecision and the dangers associated with multiplicity of analyses and outcomes. | Page 12 & 13 |
| Generalizability | 21 | [Generalizability (external validity) of the trial findings](http://www.consort-statement.org/index.aspx?o=1094). | Page 13 |
| Overall evidence | 22 | [General interpretation of the results in the context of current evidence](http://www.consort-statement.org/index.aspx?o=1095). | Page 13 & 14 |

[**www.consort-spirit.org**](http://www.consort-spirit.org)

## Supplement 2. Supplementary Table S1

|  | Subcutaneous | Intravenous |
| --- | --- | --- |
| Baseline | 0.778 (0.679 – 0.887) | 0.848 (0.757 – 0.914) |
| 3 months | 0.848 (0.749 – 0.866) | 0.776 (0.718 – 0.936) |
| 6 months | 0.791 (0.631- 0.852) | 0.813 (0.753 – 0.887) |
| Supplementary Table S1: IQR of median EQ-5D-5L index | | |

## Supplement 3: Supplementary Figure S1

**Figure S1:** Measurements of rituximab levels at different time points

For the SC group, 2 samples were taken after rituximab infusion at 6 months and therefore considered missing. For the IV group, two samples at the 2-hour measurement point and two samples at 6 months were missing. Missing samples were left out of the analyses.
